# Association between Haematological Parameters and Exposure to a Mixture of Organophosphate and Neonicotinoid Insecticides among Male Farmworkers in Northern Thailand

**DOI:** 10.3390/ijerph182010849

**Published:** 2021-10-15

**Authors:** Neeranuch Suwannarin, Tippawan Prapamontol, Tomohiko Isobe, Yukiko Nishihama, Ampica Mangklabruks, Tawiwan Pantasri, Somporn Chantara, Warangkana Naksen, Shoji F. Nakayama

**Affiliations:** 1 Ph.D. Degree Program in Environmental Science, Environmental Science Research Center, Faculty of Science, Chiang Mai University, Chiang Mai 50200, Thailand; suwannarin.ns@gmail.com; 2 Environmental and Occupational Health Sciences and Non-Communicable Diseases Center of Excellence, Research Institute for Health Sciences, Chiang Mai University, Chiang Mai 50200, Thailand; 3 Health and Environmental Risk Division, National Institute for Environmental Studies, Tsukuba 305-8506, Japan; isobe.tomohiko@nies.go.jp (T.I.); nishihama.yukiko@nies.go.jp (Y.N.); 4 Department of Internal Medicine, Faculty of Medicine, Chiang Mai University, Chiang Mai 50200, Thailand; ampica.m@cmu.ac.th; 5 Department of Obstetrics and Gynecology, Faculty of Medicine, Chiang Mai University, Chiang Mai 50200, Thailand; tawiwan.p@cmu.ac.th; 6 Environmental Science Research Center, Faculty of Science, Chiang Mai University, Chiang Mai 50200, Thailand; somporn.chantara@gmail.com; 7 Faculty of Public Health, Chiang Mai University, Chiang Mai 50200, Thailand; wnaksen@gmail.com

**Keywords:** organophosphates, dialkylphosphates, neonicotinoids, insecticides, metabolites, haematological parameters, farmworker, occupational health, environmental health

## Abstract

Exposure to insecticides may result in various health problems. This study investigated the association between haematological parameters and exposure to a mixture of organophosphate (OP) and neonicotinoid (NEO) insecticides among male farmworkers in Fang district, Chiang Mai province, northern Thailand. Concentrations of urinary dialkylphosphates, non-specific metabolites of OPs, and NEOs and their metabolites and haematological parameters were measured in 143 male farmworkers. The Bayesian kernel machine regression model was employed to evaluate the associations. Exposure to a mixture of insecticides was significantly associated with the mean corpuscular haemoglobin concentration (MCHC) when the concentrations of all the compounds and their metabolites were at the 60th percentile or higher compared with the 50th percentile. Furthermore, exposure to clothianidin (CLO) showed a decreasing association with MCHC when all the other insecticides were at their mean concentrations. CLO was the most likely compound to reduce MCHC, and this was confirmed by sensitivity analysis. These findings suggest that exposure to NEO insecticides, especially CLO, affects the haematological status relating to haemoglobin parameters.

## 1. Introduction

Organophosphates (OPs) are an important type of insecticide that has been widely used in agriculture over a long period of time [1,2]. Biomonitoring studies have shown that farmers/farmworkers and pest control operators engaged in agriculture are at high risk of exposure to OPs differing from non-occupational populations such as food contributors and consumers [3,4]. Exposure to OP insecticides can be assessed through measuring urinary common dialkylphosphate (DAP) metabolites including dimethylphosphate (DMP), dimethylthiophosphate (DMTP), dimethyldithiophosphate (DMDTP), diethylphosphate (DEP), diethylthiophosphate (DETP) and diethyldithiophosphate (DEDTP) [5,6,7]. Epidemiological studies have provided evidence that exposure to OPs, measured through DAP metabolites, increases the risk of diseases including cancer and cardiovascular disease [8,9,10,11]. However, few studies have investigated the changes of haematological indices in workers occupationally exposed to OP insecticides using DAP metabolites [12,13].

Neonicotinoids (NEOs) are another group of insecticides that have been introduced into the agricultural and household sectors over the past three decades [14]. Farmworkers and applicators employed in agriculture are at increased risk of exposure to NEOs, as compared to general populations [15,16,17,18]. Seven NEOs, namely, acetamiprid (ACE), clothianidin (CLO), dinotefuran (DIN), nitenpyram (NIT), imidacloprid (IMI), thiacloprid (THI) and thiamethoxam (THX), are commercially available and used intensively worldwide [19]. Several biomonitoring studies measured parent compounds and their metabolites to assess NEO exposure [18,20]. Currently, the most sensitive biomarkers of NEO exposure are urinary concentrations of NEO and their specific metabolites such as N-desmethyl-acetamiprid (N-dm-ACE), a metabolite of ACE, desmethyl-clothianidin (dm-CLO), a metabolite of CLO, imidacloprid-olefin (Of-IMI), a metabolite of IMI and thiacloprid-amide (THI-AM), a metabolite of THI [17,18,20,21,22]. A few animal studies have shown that exposure to NEOs can affect the blood profile and immune system [23,24].

Exposure to OPs and NEOs is a public health concern due to the associations between pesticide exposure and various adverse health outcomes, including neurodevelopmental and birth defects [25,26,27,28,29,30]. Endocrine disruption and effects on reproductive systems have also been reported [31,32,33]. Related clinical diseases might develop after haematological changes with unobservable symptoms [13]. Haematological parameters are widely used for preliminary diagnosis of diseases and include haemoglobin (Hb), haematocrit (Hct), mean corpuscular volume (MCV), mean corpuscular haemoglobin (MCH), mean corpuscular haemoglobin concentration (MCHC), red cell distribution width (RDW), red blood cell (RBC) count, white blood cell (WBC) count, platelet (PLT) count, lymphocytes (LYMs), neutrophils (NEUs), eosinophils (EOSs) and monocytes (Monos). Several epidemiological studies have established the effects of pesticide exposure on haematological alterations. For instance, agricultural farmworkers, farmers and sprayers have reduced levels of RBCs, Hb, Hct, MCV, LYMs, Monos, EOSs [34,35,36,37] and PLTs [38]. These parameters may be biomarkers of sub-clinical health effects.

Farmworkers in northern Thailand lack awareness regarding personal protective equipment (PPE) [39,40]. Moreover, farmers often do not follow the instruction and result in using higher concentrations and/or a mixture of different kinds of insecticides, leading to a higher risk of insecticide exposure [41,42]. The health effects of occupational exposures to a heterogeneous chemical mixture of insecticides are of great concern. We previously reported the association of OPs and NEOs, with serum steroid hormones among these farmworkers [43]. Still, little is known about the effects of OP exposure based on DAPs on haematological parameters [12,38,44]. To our knowledge, no human study has examined the associations between NEO exposure and blood indices. The present study investigated the associations of exposure to a mixture of OP and NEO insecticides with alterations in haematological parameters.

## 2. Materials and Methods

### 2.1. Questionnaire Data Collection

The structured questionnaire was modified from the pilot study [16]. Reliability and validity were tested as quality control (QC) for the questionnaire. Each individual was directly interviewed regarding their sociodemographic factors (age, ethnicity, education level and individual income), alcohol consumption, smoking status, work and exposure characteristics and food consumption. Trained project staff took anthropometric measurements (weight and height) and conducted the questionnaire.

### 2.2. Study Area, Study Subjects and Enrolment

This cross-sectional study was conducted in Fang district (19°54′26″ N, 99°2′26″ E), Chiang Mai province, Thailand, from June to September 2019. A total of 143 male farmworkers who could conveniently participate in the study were recruited based on the following screening criteria: 18–40 years of age and worked at least 3 days per week in the areas. Prior to enrolment, the purpose of the study was described to all subjects. The participants signed an informed consent form prior to face-to-face interviews using a structured questionnaire and collection of urine and blood.

### 2.3. Urine Collection

Spot urine samples were self-collected in a 50 mL provided container, aliquoted into 10 and 5 mL cryotubes (Greiner Bio-One Co. Ltd., Tokyo, Japan), kept at −20 °C at the laboratory field site prior to being transferred on dry ice to the Toxicology Laboratory at the Research Institute for Health Sciences (RIHES), Chiang Mai University and stored at −20 °C until analysed. Additionally, 4 mL of each urine sample was shipped on dry ice to the Health and Environmental Risk Division, National Institute Research for Environmental Studies (NIES), Tsukuba, Japan and stored at −80 °C until analysed.

### 2.4. Blood Collection

Non-fasting venous blood samples (25 mL) were drawn from male participants in the morning between 05:00 a.m. and 12:00 p.m. Approximately 3 mL of whole blood was aliquoted into a K_2_EDTA-coated vacutainer tube. The samples were transported in cold conditions to the Chiang Mai R.I.A. Laboratory Clinic, a certified professional clinical laboratory, and analysed for various haematological parameters on the same day as blood collection.

### 2.5. Urinary OP Metabolite Analysis

Urinary DAP metabolites including DMP, DMTP, DEDTP, DEP, DETP and DEDTP were analysed by gas chromatography with a flame photometric detector after derivatisation according to the previously established method using an Agilent 7890-B GC system (Agilent Technologies, Inc., Santa Clara, CA, USA) [45]. The procedure was briefly described in our previous study [16].

### 2.6. Urinary NEO and Their Metabolite (NEO/m) Analysis

Urinary NEO/m, including ACE, CLO, DIN, flonicamid (FLN), IMI, NIT, sulfoxaflor (SUF), THI, THX, dm-CLO, N-dm-ACE, Of-IMI and THI-AM were measured by liquid chromatography-tandem mass spectrometry according to our previous study using a Nexera liquid chromatography system coupled to a Triple Quad 8060 mass spectrometer (Shimadzu Corporations, Kyoto, Japan) [16].

### 2.7. Haematological Parameter Analysis

A total of 13 complete blood count parameters were examined using a Mindray BC-5300 Auto Haematology Analyzer (Shenzhen Mindray Bio-Medical Electronics Company Limited, Shenzhen, China). The haematological parameters studied were Hb, Hct, MCV, MCH, MCHC, RDW, RBC count, WBC count, NEUs, LYMs, EOSs, Monos and PLT count.

### 2.8. QC and Quality Assurance

#### 2.8.1. Urinary OP Metabolite Analysis

Urine samples were pooled from anonymous non-farming volunteers and used as QC samples. The QC urine samples were analysed for reproducibility precision. The procedure was described in our previous study [43]. Proficiency testing materials from the German External Quality Assessment Scheme (G-EQUAS) were analysed as part of the quality assurance of the method. Overall, the reported values fell well within the tolerance ranges (Supplementary Materials, Table S1).

#### 2.8.2. Urinary NEO/m Analysis

Urine samples were collected from pregnant volunteers from Japan and pooled as QC samples. The QC samples were analysed as part of quality assurance and the results were recorded in a Shewhart control chart ($\overset{¯}{X}$-R control chart) based on ISO 7870. Duplicate analyses of samples were performed every analytical batch of 40 samples during measurement. The procedure was described in our previous study [43].

### 2.9. Statistical Analysis

The subject-related sociodemographic factors, work and exposure characteristics and food consumption were reported as the frequency distribution or mean ± standard deviation (SD). Urinary concentrations of DAPs and NEO/m were normalised relative to specific gravity (SG) in the same samples to adjust for urine dilution. These concentrations were, therefore, reported as ng/mL. Concentrations were corrected for SG using the following adapted formula [46]:

P_c_ = P [(SG_Med_ − 1/SG_Meas_ − 1)] (1)

where P_c_ is the SG-corrected metabolite concentration, P is the observed metabolite concentration, SG_Med_ is the median SG of all samples tested in the study and SG_Meas_ is the measured SG of the individual urine sample. The concentrations of DAPs and NEO/m were assessed to check normality using the Kolmogorov–Smirnov test and log10-transformed to obtain normal distributions before statistical analyses. Summary statistics were computed using the NADA package (version 1.6-1.1) in the statistical R software. Geometric means (GMs) and geometric standard deviations (GSDs) were calculated for analytes detected in >50% of samples. The range and detection frequency were also determined. Subject-related sociodemographic characteristics of participants with missing data were computed by the multivariate imputation by chained equations (MICE) method, with 10 imputations and 10 iterations using MICE package (version 3.13.0). In addition, urinary concentrations below the method detection limit (MDL) were imputed with quantile regression approach for left-censored missing (QRILC) for subsequent statistical analysis.

Bayesian kernel machine regression (BKMR) analysis was used to investigate the associations between OP and NEO exposure and haematological parameters [47,48]. The BKMR model was used to estimate the health effects of exposure to insecticide mixtures, in which the health outcome was regressed on a flexible function of the mixture components using a Gaussian kernel function with the bkmr package (version 0.2.0.9000) [47]. The urinary DAP and NEO/m concentrations detected in more than 25% of samples were incorporated into the model. A hierarchical variable selection method was conducted with 10,000 iterations by a Markov chain Monte Carlo algorithm due to the highly correlated nature of the mixture of chemicals. Based on the chemical structures of the insecticide mixtures, DAPs including DMP, DMTP, DMDTP, DEP, DETP and DEDTP were categorised into Group 1, while NEO/m including ACE, CLO, THX, IMI, SUF, N-dm-ACE, dm-CLO and Of-IMI were categorised into Group 2. The general modelling framework was considered using the following formula:

Y_i_ = *h* [Group_1_ = (DMP, DMTP, DMDTP, DEP, DETP, DEDTP), Group_2_ = (ACE, CLO, THX, IMI, SUF, N-dm-ACE, dm-CLO and Of-IMI)] + βiX_i_ (2)

where Y_i_ is the health endpoint (haematological parameters), *h*(…) is the exposure–response function assuming it is non-linear and non-additive among the mixture components, X_i_ is the vector of covariates assumed to have a linear relationship with the outcome and β is the vector of coefficients. A hierarchical variable selection method was used to calculate the group posterior inclusion probability (groupPIP) representing the probability for a mixture group, which was included in the final model after 10,000 iterations. Based on groupPIP, the conditional posterior inclusion probability (condPIP) was calculated, which represented the probability that a particular insecticide or its metabolites within the group was included in the model. A PIP threshold of 0.5 is usually used to determine whether it is important [49]. The BKMR model was adjusted for age, ethnicity, education, individual income, smoking status, alcohol consumption, body mass index (BMI), work and exposure characteristics, behaviour during and after working and food consumption.

In addition, sensitivity analysis was performed to evaluate the robustness of the BKMR results using a Bayesian generalised linear regression model (GLM). The GLM was adjusted forage, BMI, ethnicity, education, smoking status, alcohol consumption, work and exposure characteristics, behaviour during and after working and food consumption. The analysis was performed using brms package (version 2.16.1). All statistical analyses were performed using the statistical software R version 4.1.1 [50].

## 3. Results

### 3.1. Sociodemographic Characteristics of the Study Participants

The sociodemographic characteristics of the study participants were described in our previous study [43]. Briefly, the study participants had a mean age of 30.1 (SD = 5.8) years. Their mean BMI was 23.5 (4.3) kg/m^2^ and half of the participants (50%) were a normal weight. Only 7% of the participants were Northern Thai, and most of the participants (92%) self-identified as other ethnicities. In total, 57% of the participants had no formal education and 28% had completed primary school. 

### 3.2. Urinary DAP and NEO/m Concentrations

Table 1 shows urinary concentrations of DAPs and NEO/m among male farmworkers. DAP metabolites were detected in the urine samples of participants, with the detection frequency ranging from 25.2% (DMDTP) to 100.0% (DEP). The GM concentration of DETP was highest (23.9 ng/mL). The detection frequency of NEO/m ranged from 0.7% (THI-AM) to 99.3% (IMI and N-dm-ACE), and the GM concentration of IMI was highest (17.4 ng/mL). The maximum concentration of a NEO/m in a single sample was that of Of-IMI (253 ng/mL), a metabolite of IMI.

### 3.3. Haematological Parameters among Male Farmworkers 

The characteristics of haematological parameters among male farmworkers are described in Table 2. The average haematological parameters of all samples were normal. However, approximately 17% of male farmworkers had a Hb value below the normal range. Another RBC test showed that Hct, MCV, RDW and the RBC count were outside the normal ranges in 15%, 16%, 1% and 13% of male farmworkers, respectively, while MCH and MCHC were below the normal ranges in 34% and 5% of farmworkers, respectively. A total of 25% and 6% of farmworkers had abnormal WBC counts and LYM levels, respectively. In addition, 9% of male farmworkers had a PLT count below the normal range. NEU, EOS and Mono levels were within the normal ranges (100%).

### 3.4. The Associations between DAP and NEO/m Concentrations and Haematological Parameters

The log-transformed concentration of each compound was treated as a continuous variable and fitted with the BKMR model to assess the joint effect of exposure to the mixture of insecticides on haematological parameters. DAPs including DMP, DMTP, DMDTP, DEP, DETP and DEDTP were categorised into Group 1, while NEO/m including ACE, CLO, THX, IMI, SUF, N-dm-ACE, dm-CLO and Of-IMI were categorised into Group 2. The posterior inclusion probabilities derived from the BKMR model for the two groups (groupPIP) and each chemical (condPIP) in the MCHC model are provided in Table 3. The groupPIP of DAPs, Group 1, was low while the groupPIP of NEO/m, Group 2, was extremely high at 0.996, which indicated the NEO/m group had a dominant effect on MCHC. The condPIP of CLO was highest at 0.936, which suggested CLO was the most influential compound among the NEO/m that was associated with the MCHC. Meanwhile, the condPIPs of each DAP metabolite were evenly low.

**Figure 1:**
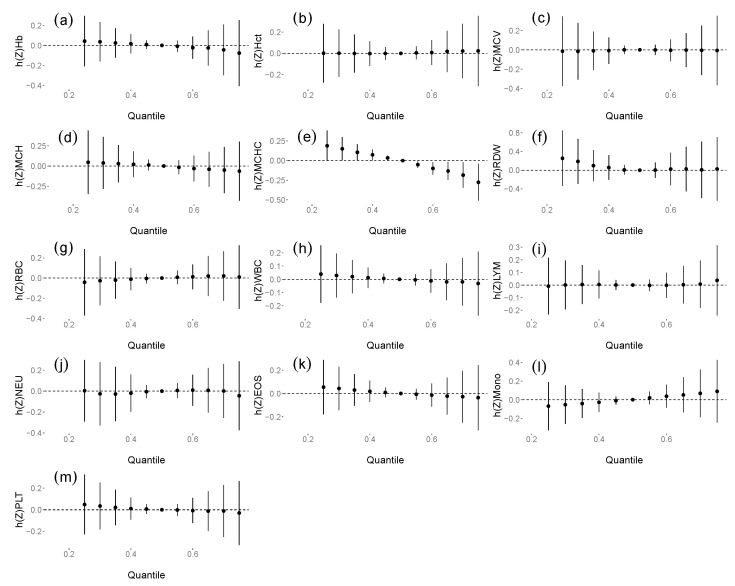
Overall effect (95% CI) of exposure to the insecticide mixture on haemoglobin (Hb) (**a**), haematocrit (Hct) (**b**), mean corpuscular volume (MCV) (**c**), mean corpuscular haemoglobin (MCH) (**d**), mean corpuscular haemoglobin concentration (MCHC) (**e**), red cell distribution width (RDW) (**f**), red blood cell (RBC) count (**g**), white blood cell (WBC) count (**h**), lymphocyte (LYM) (**i**), neutrophil (NEU) (**j**), eosinophil (EOS) (**k**), monocyte (Mono) (**l**) and platelet (PLT) count (**m**) determined by the Bayesian kernel machine regression (BKMR) model when the concentrations of all the compound and their metabolites were at a particular percentile compared with when the concentrations of all the compound and their metabolites were at the 50th percentile. The models were adjusted for age, ethnicity, education, individual income, smoking status, alcohol consumption, body mass index (BMI), work and exposure characteristics, behaviour during and after working, food consumption and food items consumed in the past month.

The overall associations between exposure to the DAP and NEO/m mixture on haematological parameters are shown in Figure 1. MCHC significantly decreased when the concentrations of all the compounds and their metabolites were at the 60th percentile or higher compared with the 50th percentile, indicating a negative association with MCHC. No statistically significant association was found between exposure to the insecticide mixture and other haematological parameters; however, there was a decreasing trend in the Hb, MCH, WBC and EOS models and an increasing trend in the Mono model. We investigated the trends of exposure–response functions of NEO/m and DAPs. The results are shown in Figure S1. When all other chemicals were at their median levels in the univariate exposure-response function, CLO showed a decreasing association with MCHC.

### 3.5. Sensitivity Analysis

The results from the BKMR model were confirmed by GLM analysis. The association between urinary concentrations of CLO and MCHC determined by GLM is provided in Table 4. The GLM also showed a negative association between CLO and MCHC (see also Figures S1 and S2).

## 4. Discussion

The current study found that urinary DAP and NEO/m concentrations of male farmworkers engaged in agriculture in Chiang Mai province were higher than in our previous study [16]. The BKMR model revealed the overall effect of exposure to a mixture of insecticides, namely, OPs and NEOs, on haematological indices among male farmworkers. Furthermore, in the MCHC model, the groupPIP of NEO/m was very high and CLO had a high condPIP within the NEO/m group. Moreover, CLO showed a negative relationship with MCHC when all the other chemicals were fixed at their median levels. The sensitivity analysis supported the consistency of the results.

### 4.1. Urinary DAP and NEO/m Concentrations

The current study shows that male farmworkers were exposed to OP insecticides. The urinary concentration of DETP was the highest among DAP metabolites. This was consistent with our previous study; however, farmworkers in this study tended to have higher concentrations of DEP, DETP and DEDTP than in the previous study [16]. This can be explained by the fact that urine samples were collected from June to September, which is when crop cultivation starts, in the present study, but in February, which is after harvesting, in the previous study [41,42]. Similarly, there were seasonal differences in previous studies in Japan and the Netherlands, but they investigated dimethyl moieties instead of diethyl moieties, which are more potent [51,52,53]. By contrast, a previous study of Thai children reported no significant difference in DAPs between seasons in which pesticide use was low and high [27]. Specific spatial determinants might contribute to this difference. Another explanation for the differences in diethyl moiety concentrations might be due to type of cultivation crops notably citrus fruit in Fang district, which farmworkers may apply larger quantities of pesticides than in other crops, i.e., cut flowers, cultivated in the previous study’s another study site.

Male farmworkers in the current study were commonly exposed to NEOs. The detection frequencies were comparable to our previous study, but the GM concentrations in the present study were higher than those in the previous study: N-dm-ACE (19.2 vs. 7.3 µg/g Cr), IMI (25.7 vs. 8.7 µg/g Cr), THX (14.0 vs. 4.3 µg/g Cr), CLO (10.7 vs. 2.4 µg/g Cr) and Of-IMI (5.8 vs. 2.6 µg/g Cr) [16]. In addition, the GM concentration of IMI was higher than that of Chinese pesticide applicators both before and after pesticide application (2.8 and 10.5 µg/g Cr, respectively) [15]. There were no other previous studies of urinary concentrations of NEO/m most frequently detected in the present study among pesticide sprayers or farmworkers. Concentrations of N-dm-ACE, CLO, THX and Of-IMI were higher than observed in East Asia and the U.S. [54,55,56,57,58,59,60]. Our study detected multiple NEO insecticides with high detection rates and concentrations in urine samples. This is the first study of its kind and, therefore, further research is warranted.

### 4.2. Associations between Insecticide Exposure and Haematological Parameters

Exposure to pesticides was reported to have harmful effects on haematological parameters, including Hb, Hct, MCV, MCH, MCHC, RBCs, WBCs, LYMs and PLTs [13,34,61,62,63,64]. Changes in the complete blood count due to NEO exposure have not been established. For the first time, we reported urinary concentrations of NEO/m and their associations with haematological parameters. Less than 34% of male farmworkers had MCH levels lower than the clinical reference range and the mean levels of all parameters were within the normal ranges, consistent with previous studies [37,65]. Previous studies revealed that MCV, MCH and MCHC were significantly lower in farmers than in the control group, suggesting that farmers are at risk of developing anaemia [65]. However, the levels of these parameters in the control group in that study were similar to those in our study. A study of farmers in Iran reported Hb, Hct and RBC levels similar to those in our study. However, they found that Hb, Hct and RBC levels were increased among farmers exposed to pesticides, but MCV was decreased [66]. These discrepancies in haematological changes are probably due to differences in the pattern of pesticide use, frequency and dose of exposure and study design [66,67].

Erythropoiesis occurs in the bone marrow and is regulated by a negative feedback loop whereby tissue hypoxia stimulates the secretion of erythropoietin by the kidneys [68]. In our study, we found a negative association between CLO and MCHC. MCHC indicates the average concentration of Hb per unit volume of RBCs and correlates the Hb content with the packed cell volume [69]. In addition, we found a decreasing trend of Hb with THX and CLO when all other chemicals were at their median levels in the univariate exposure-response function (data not shown). A recent study reported that THX poisoning causes acute kidney injury in proximal tubules by inhibiting the α7 nicotinic receptor [70,71]. CLO was reported to be a specific metabolite of THX [72], and the urinary concentrations of THX and CLO were strongly correlated [60]. Based on these findings, we hypothesized that exposure to CLO can cause kidney injury in the same manner as THX, leading to less secretion of EPO and consequently less production of Hb in RBCs. Although limited human studies have reported data, a few animal studies provided evidence that NEOs affect blood cell profiles [23]. For instance, Ghaffar et al. found that the levels of RBCs and Hb decreased after exposure to THX in fresh-water fish, suggesting that poor efficiency of fresh-water fish to deliver enough amount of oxygen to other tissues [73]. Acute exposure to IMI changes RBCs, Hct and the erythrocyte sedimentation rate in female mice [74], whereas exposure to a sublethal IMI concentration decreases the number of erythrocytes in Nile tilapia fish [24]. RBC, Hb and Hct levels were decreased in Wistar rats treated with a high dose of ACE [75]. Moreover, exposure to THX elevates the NEU-to-LYM and NEU-to-leukocyte ratios, while exposure to CLO causes anaemia in frogs, suggesting that exposure to high concentrations of CLO and THX exerts haematotoxic effects [23]. Another possible explanation for the decreases in Hb, MCH and MCHC is the thalassemia trait in our study population due to the high prevalence of thalassemia in hill tribes in Southeast Asia, notably northern Thailand [76,77].

### 4.3. Limitations

Although our study reports novel findings regarding the association of exposure to NEO insecticides with MCHC, it has some limitations. First, OPs are rapidly metabolised and their half-lives are relatively short; therefore, the concentrations of DAPs reflect recent exposure, predominantly in the previous few days (24–72 h) [78,79]. However, DAP concentrations reflect exposure to multiple OP insecticides and are widely used [79,80,81]. NEOs have shorter half-lives than their metabolites, and we included NEO/m to represent a wide range of NEO exposures [82]. However, interclass correlation coefficients ranged from 0.09 to 0.42 for measurements of NEOs, including CLO, IMI, THX and N-dm-ACE, indicating this approach was inaccurate for estimating NEO exposure over a month [59]. In addition, we did not include the baseline of haematological data among general populations, in contrast with some previous studies [83,84]. However, the haematological parameters such as Hb, Hct, MCV and MCH of non-farmworkers (*n* = 18) were comparable to those of the farmworkers (*n* = 143) but the exposure concentrations were significantly different (unpublished data). This is the first study to report the effects of OP and NEO exposure on the haematological status of male farmworkers. Further studies should perform follow-up to compare whether the effects differ between periods in which exposure to pesticides is high and low.

## 5. Conclusions

The current study revealed that exposure to a mixture of DAP and NEO/m was significantly associated with MCHC. Furthermore, CLO was the compound that influenced MCHC. We assume that human exposure to NEO insecticides causes toxicity and affects haematological parameters relating to RBC indices, including Hb, MCH and MCHC. Although these findings are consistent with some animal studies, further studies are warranted to confirm them in human populations.

## Figures and Tables

**Table 1:** Urinary concentrations of dialkylphosphates (DAPs) and neonicotinoids and their metabolites (NEO/m) among male farmworkers (*n* = 143).

Compound (ng/mL)	MDL	>MDL (%)	GM (GSD)	Range
DAPs:				
DMP	5.0	28.7	−	<MDL–73.4
DMTP	1.0	44.8	−	<MDL–134
DMDTP	0.5	25.2	−	<MDL–398
DEP	1.0	100.0	20.7 (4.5)	<MDL–5678
DETP	0.125	99.3	23.9 (4.2)	<MDL–445
DEDTP	0.25	79.0	9.3 (6.1)	<MDL–386
NEO/m:				
ACE	0.0011	46.9	−	<MDL–1.5
CLO	0.0009	96.5	7.4 (3.6)	0.01–14.6
DIN	0.003	16.1	−	<MDL–1.0
FLN	0.003	16.8	−	0.004–0.02
IMI	0.007	99.3	17.4 (5.5)	0.02–24.6
NIT	0.009	3.5	−	<MDL–0.3
THI	0.007	4.9	−	<MDL–0.009
THX	0.002	97.2	9.1 (4.9)	0.004–75.8
SUF	0.002	35.7	−	<MDL–0.3
dm-CLO	0.01	29.4	−	<MDL–4.1
N-dm-ACE	0.007	99.3	15.8 (3.7)	<MDL–18.1
Of-IMI	0.03	64.3	5.1 (5.3)	0.12–253
THI-AM	0.2	0.7	−	<MDL–0.04

Abbreviations: MDL, method detection limit; ng/mL, nanograms per millilitre; GM, geometric mean; GSD, geometric standard deviation; DAP, dialkylphosphate; DMP, dimethylphosphate; DMTP, dimethylthiophosphate; DMTDP, dimethyldithiophosphate; DEP, diethylphosphate; DETP, diethylthiophosphate; DETDP, diethyldithiophosphate; NEO/m, neonicotinoids and their metabolites; ACE, acetamiprid; CLO, clothianidin; DIN, dinotefuran; FLN, flonicamid; IMI, imidacloprid; NIT, nitenpyram; THI, thiacloprid; THX, thiamethoxam; SUF, sulfoxaflor; dm-CLO, desmethyl-clothianidin; N-dm-ACE, N-desmethyl-ACE; Of-IMI, imidacloprid-olefin; THI-AM, thiacloprid-amide.

**Table 2:** Characteristics of haematological parameters among farmworkers (*n* = 143).

Haematological Parameter	Mean ± SD	Min–Max	*n* (%) Normal	*n* (%) below the Range	*n* (%) above the Range
Hb (g/dL)		9.8–17.8	119 (83.2)	24 (16.8)	0 (0)
Hct (%)	46.2 ± 3.7	32.1–53.6	122 (85.3)	2 (1.4)	19 (13.2)
MCV (fL)	87.4 ± 8.0	53.5–107.0	119 (83.2)	20 (13.4)	4 (2.8)
MCH (pg)	28.6 ± 3.0	16.5–35.2	95 (66.4)	48 (33.6)	0 (0)
MCHC (g/dL)	32.7 ± 1.0	29.8–34.8	136 (95.1)	7 (4.9)	0 (0)
RDW (%)	12.0 ± 1.2	1.9–17.2	141 (98.6)	1 (0.7)	1 (0.7)
RBC count (×10^6^/µL)	5.3 ± 0.5	3.9–7.3	124 (86.7)	6 (4.2)	13 (9.1)
WBC count (cells/µL)	6300 ± 1710	2500–12,700	107 (74.8)	32 (22.3)	4 (2.8)
NEU (%)	60.4 ± 5.0	48.0–69.0	143 (100)	0 (0)	0 (0)
LYM (%)	37.2 ± 5.5	12.0–51.0	135 (94.4)	1 (0.7)	7 (4.9)
EOS (%)	1.0 ± 0.9	0–6.0	143 (100)	0 (0)	0 (0)
Mono (%)	1.1 ± 0.8	0–4.0	143 (100)	0 (0)	0 (0)
PLT count (cells/µL)	229,000 ± 67,300	47,000–399,000	130 (90.9)	13 (9.1)	0 (0)

Abbreviations: Hb, haemoglobin; g/dL, grams per decilitre; Hct, haematocrit; MCV, mean corpuscular volume; fL, femtolitre; MCH, mean corpuscular haemoglobin; pg, picogram; MCHC, mean corpuscular haemoglobin concentration; RDW, red cell distribution width; RBC, red blood cell; µL, microlitre; WBC, white blood cell; NEU, neutrophil; LYM, lymphocyte; EOS, eosinophil; Mono, monocyte; PLT, platelet, SD, standard deviation; Min, minimum; Max, maximum. Note: normal laboratory reference ranges of haematological parameters are as follows: Hb, 14–18 g/dL for men; Hct, 36–50%; MCV, 80–100 fL; MCH, 28–36 pg; MCHC, 31–37 g/dL; RDW, 10.0–16.5%; WBC count, 5000–10,000 cells/µL; RBC count, 4.5–6.0 × 10^6^/µL; platelets, 140,000–400,000 cells/µL; neutrophils, 40–70%; lymphocytes, 20–45%; eosinophils, 0–6% and monocytes, 0–10%; The reference values for haematological parameters were provided by the Chiang Mai R.I.A. Laboratory Clinic, a certified professional clinical laboratory.

**Table 3:** Posterior inclusion probabilities (PIPs) for group inclusion and conditional inclusion in mean corpuscular haemoglobin concentration (MCHC) models using the Bayesian kernel machine regression (BKMR) model.

Variable	Group	GroupPIP	CondPIP
DAPs:			
DMP	1	0.241	0.256
DMTP	1	0.241	0.043
DMDTP	1	0.241	0.122
DEP	1	0.241	0.081
DETP	1	0.241	0.258
DEDTP	1	0.241	0.239
NEO/m:			
ACE	2	0.996	0.000
THX	2	0.996	0.024
CLO	2	0.996	0.936
IMI	2	0.996	0.000
SUF	2	0.996	0.016
N-dm-ACE	2	0.996	0.000
dm-CLO	2	0.996	0.023
Of-IMI	2	0.996	0.000

The models were adjusted for age, ethnicity, education, individual income, smoking status, alcohol consumption, BMI, work and exposure characteristics, behaviour during and after working, food consumption and food items consumed in the past month. Abbreviations: DAP, dialkylphosphate; DMP, dimethylphosphate; DMTP, dimethylthiophosphate; DMTDP, dimethyldithiophosphate; DEP, diethylphosphate; DETP, diethylthiophosphate; DETDP, diethyldithiophosphate; NEO/m, neonicotinoids and their metabolites; ACE, acetamiprid; THX, thiamethoxam; CLO, clothianidin; IMI, imidacloprid; SUF, sulfoxaflor; N-dm-ACE, N-desmethyl-ACE; dm-CLO, desmethyl-clothianidin; Of-IMI, imidacloprid-olefin; GroupPIP, group posterior inclusion probability; CondPIP, conditional posterior inclusion probability.

**Table 4:** Bayesian generalised linear regression analysis of the association between concentrations of clothianidin (CLO) and the mean corpuscular haemoglobin concentration (MCHC) among male farmworkers (*n* = 143).

Dependent Variable	Independent Variable	Coefficient B (95% CI)	Conditional R^2^
MCHC	CLO	−0.43 (−0.7, −0.16)	0.463

Abbreviations: MCHC, mean corpuscular haemoglobin concentration; CLO, clothianidin; B, unstandardised partial regression coefficient; CI, confidence interval; R^2^, coefficient of determination.

## Supplementary Materials

The following are available online at https://www.mdpi.com/article/10.3390/ijerph182010849/s1, Table S1: urinary dialkylphosphate (DAP) concentrations in the German External Quality Assessment Scheme (G-EQUAS) materials batch no. 64/2019, Table S2: work and pesticide exposure characteristics and food consumption of the study participants, Figure S1: univariate exposure–response function (95% CI) between selected chemical concentrations and mean corpuscular haemoglobin concentrations (MCHCs) when the concentrations of other chemicals were fixed at the median values using the Bayesian kernel machine regression (BKMR) model. Figure S2. The association between clothianidin (CLO) and mean corpuscular haemoglobin concentrations (MCHCs) using a Bayesian generalised linear regression model.
