# Supplementary material for: Association between Haematological Parameters and Exposure to a Mixture of Organophosphate and Neonicotinoid Insecticides among Male Farmworkers in Northern Thailand
Source: Int J Environ Res Public Health. 2021 Oct 15;18(20):10849. doi: 10.3390/ijerph182010849 (PMC8535230; doi:10.3390/ijerph182010849)
Supplement: Supplementary file 1 [file ijerph-18-10849-s001.zip › ijerph-1334459-supplementary.pdf]

International Journal of
Environmental Research and Public Health

MDPI

Supplementary materials

Association between Haematological Parameters and Exposure to a mixture of Organophosphate and Neonicotinoid Insecticides among Male Farmworkers in Northern Thailand

Table S1. Urinary dialkylphosphate (DAP) concentrations in the German External Quality Assessment Scheme (G-EQUAS) materials batch no. 64/2019.

Analyte	Level A Reported value	Reference value	Tolerance range	Level B Reported value	Reference value	Tolerance range
DMP	18.34	18.12	12.51–23.73	58.73	71.79	55.20–88.38
DMTP	13.61	11.98	7.69–16.27	28.46	30.61	22.45–38.77
DMDTP	2.64	2.91	1.86–3.96	3.9	4.61	3.11–6.11
DEP	4.74	4.98	3.27–6.69	37.41	45.64	32.80–58.48
DETP	16.92	15.33	9.54–21.12	86.05	87.7	70.75–104.65
DEDTP	0.88	0.68	0.38–0.98	2.53	2.03	1.34–2.72

Note: Analyte concentrations are reported as μg/L (or ng/mL). Abbreviations: DMP, dimethylphosphate; DMTP, dimethylthiophosphate; DMTDP, dimethyldithiophosphate; DEP, diethylphosphate; DETP, diethylthiophosphate; DETDP, diethyldithiophosphate.

Table S2. Work and pesticide exposure characteristics and food consumption of the study participants.

Variable	n (%) Men (*n*=143)
Total number of years spent as a farmworker	
Mean ± SD	8.9 ± 6.4
≤ 5 years	51 (35.7)
6–10 years	39 (27.3)
11–20 years	38 (26.6)
> 20 years	8 (5.5)
NA	7 (4.9)
Status of farmworker	
Working on own farm or family farm; owner	65 (45.4)
Working on another person's farm; permanent labourer	63 (44.1)
Working on a rented farm	15 (10.5)
NA	1 (0.7)
Living in proximity to the agricultural field	
Yes	83 (58.0)
No	59 (41.3)
Hours/day spent working in the field	
Mean ± SD	8.1 ± 1.8
NA	2 (1.4)
Days/week spent working in the field	
Mean ± SD	5.5 ± 1.7

NA	3 (2.1)
Frequency of pesticide use	
Once per month	12 (8.4)
Twice per month	10 (6.9)
Thrice per month	13 (9.1)
Once per week	47 (32.9)
2–3 times per week	24 (16.8)
4–5 times per week	4 (2.8)
> 5 times per week	16 (11.2)
Don't know	17 (11.9)
Last pesticide use	
Three days prior to urine collection and the day of urine collection	66 (46.1)
Two weeks ago	24 (16.8)
One month ago	5 (3.5)
> One month ago	14 (9.8)
Don't know	34 (23.8)
Number of crops cultivated	
1	88 (62.0)
≥ 2	55 (38.0)
Type of crop cultivated (multiple choice)	
Tangerine	98 (68.5)
Corn	38 (26.6)
Lychee	10 (6.9)
Longan	7 (4.9)
Red bean	37 (25.9)
Strawberry	7 (4.9)
Rice	3 (2.1)
Mango	1 (0.7)
Cabbage	5 (3.5)
Garlic	3 (2.1)
Rambutan	1 (0.7)
Watermelon	0 (0.0)
Passion fruit	1 (0.7)
Pineapple	1 (0.7)
Pesticide-related activities	
Mixed pesticides	
Yes	70 (48.9)
No	72 (50.3)
NA	1 (0.7)
Sprayed pesticides	
Yes	110 (77.9)
No	32 (22.4)
NA	1 (0.7)
Scattered seeds	
Yes	32 (22.4)
No	110 (77.9)
NA	1 (0.7)

Harvested crops	
Yes	103 (72.0)
No	39 (27.3)
NA	1 (0.7)
Received training regarding pesticide use or personal protective equipment	
Yes	25 (17.5)
No	118 (82.5)
Behaviour during and after working	
Eat or drink while in the field	
Yes	113 (79.0)
No	30 (21.0)
Smoke while in the field	
Yes	50 (35.0)
No	93 (65.0)
Wash hands after work	
Yes	141 (98.6)
No	2 (1.4)
Shower before shifting to other activities after work	
Yes	77 (53.8)
No	66 (46.2)
Wear the same clothes on two consecutive days	
Yes	60 (42.0)
No	83 (58.0)
Sort working clothes and usual clothes for laundry	
Yes	96 (67.1)
No	47 (32.9)
Take off shoes before entering the home	
Yes	142 (99.3)
No	1 (0.7)
Wash feet before entering the home	
Yes	126 (88.1)
No	17 (11.2)
Store pesticides at home	
Yes	50 (35.0)
No	93 (65.0)
Vegetable sources	
Homegrown	53 (37.1)
Local village shop	76 (53.1)
Local village market	10 (7.0)
Food truck	2 (1.4)
Supermarket	2 (1.4)
Homegrown vegetable consumption	
Yes	86 (60.1)
No	57 (39.9)
For farmworkers who answered 'yes' they consumed homegrown vegetables	
Homegrown vegetables treated with pesticides	

Yes	19 (22.1)
No	67 (77.9)
Fruit sources	
Homegrown	38 (26.6)
Local village shop	69 (48.2)
Local village market	28 (19.6)
Food truck	0 (0.0)
Supermarket	8 (5.6)
Homegrown fruit consumption	
Yes	51 (35.7)
No	92 (64.3)
For farmworkers who answered 'yes' they consumed homegrown fruits	
Homegrown fruits treated with pesticides	
Yes	28 (54.9)
No	23 (45.1)
Drinking water	
Tap	23 (16.0)
Bottled	38 (26.6)
Well	28 (19.6)
Stream	54 (37.8)
Food items consumed in the past month	
Lean meat products	
Yes	141 (98.6)
No	1 (1.4)
Offal: pork or chicken	
Yes	121 (84.6)
No	22 (15.4)
Processed meat products: sausage/Thai sausage/bacon/ham/fermented pork/Vietnamese pork sausage	
Yes	107 (74.8)
No	36 (25.2)
Fatty meat products	
Yes	139 (97.2)
No	4 (2.8)
Dried meat products	
Yes	97 (67.8)
No	46 (32.2)
Seafood: fish/squid/shrimp/crab/shellfish	
Yes	59 (41.3)
No	84 (58.7)
Eggs: chicken and duck eggs	
Yes	142 (99.3)
No	1 (0.7)
Milk products	
Yes	114 (79.7)
No	29 (20.3)

High-fat foods: deep-fried food, i.e., fried chicken/fried meatballs	
Yes	131 (91.6)
No	12 (8.4)
Curry containing coconut milk or Thai desserts containing coconut milk	
Yes	74 (51.7)
No	69 (48.3)
Bakery products	
Yes	82 (57.3)
No	61 (42.7)
Snacks	
Yes	105 (73.4)
No	38 (26.6)
Fast food or branded foods: pizza/hamburger/KFC/sandwich	
Yes	34 (23.8)
No	109 (76.2)
Fresh fruits and vegetables	
Yes	136 (95.1)
No	7 (4.9)
Soft drinks	
Yes	132 (92.3)
No	11 (7.7)
Ready meals	
Yes	134 (93.7)
No	9 (6.3)
Dietary supplements: minerals/vitamins	
Yes	52 (36.4)
No	91 (63.6)
Drugs and supplements	
Yes	20 (14.0)
No	123 (86.0)

Abbreviations: SD, standard deviation. Results are reported as mean ± SD or n (%).

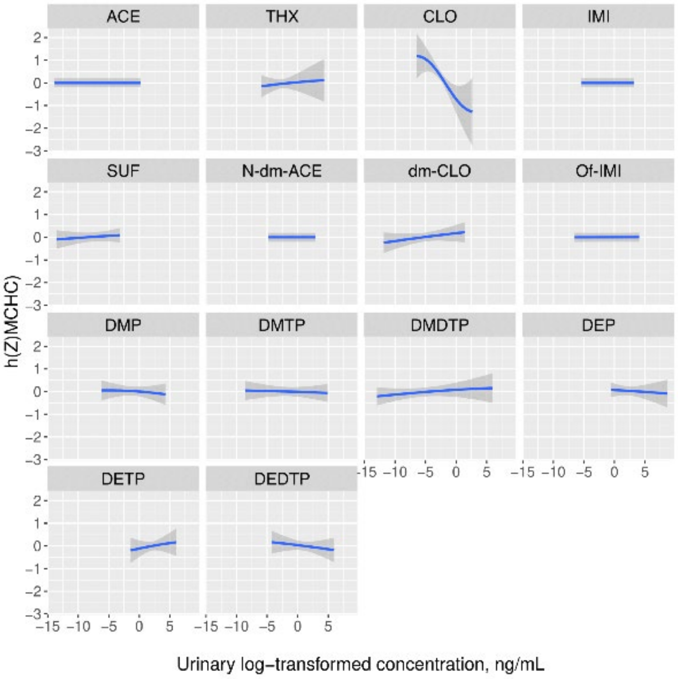

Figure S1. Univariate exposure–response function (95% CI) between selected chemical concentrations and mean corpuscular haemoglobin concentrations (MCHCs) when the concentrations of other chemicals were fixed at the median values using the Bayesian kernel machine regression (BKMR) model. The models were adjusted for age, ethnicity, education, individual income, smoking status, alcohol consumption, body mass index (BMI), work and exposure characteristics, behaviour during and after working, food consumption and food items consumed in the past month. Abbreviations ACE, acetamiprid; THX, thiamethoxam; CLO, clothianidin; IMI, imidacloprid; SUF, sulfoxaflor; N-dm-ACE, N-desmethyl-ACE; dm-CLO, desmethyl-clothianidin; Of-IMI, imidacloprid-olefin; DMP, dimethylphosphate; DMTP, dimethylthiophosphate; DMTDP, dimethyldithiophosphate; DEP, diethylphosphate; DETP, diethylthiophosphate; DETDP, diethyldithiophosphate.

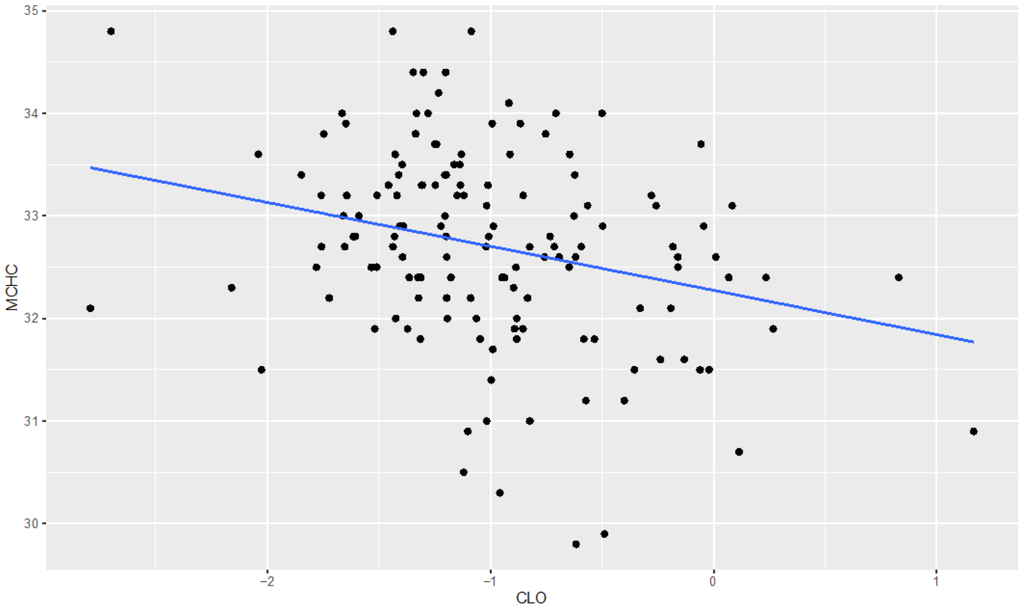

Figure S2. The association between clothianidin (CLO) and mean corpuscular haemoglobin concentrations (MCHCs) using a Bayesian generalised linear regression model. The models were adjusted for age, ethnicity, education, individual income, smoking status, alcohol consumption, body mass index (BMI), work and exposure characteristics, behaviour during and after working, food consumption and food items consumed in the past month.
